# Data-driven subtypes of major depressive disorder: a systematic review

**DOI:** 10.1186/1741-7015-10-156

**Published:** 2012-12-04

**Authors:** Hanna M van Loo, Peter de Jonge, Jan-Willem Romeijn, Ronald C Kessler, Robert A Schoevers

**Affiliations:** 1 Department of Psychiatry, University Medical Center Groningen, Hanzeplein 1, Groningen, 9713 GZ, The Netherlands; 2 Faculty of Philosophy, University of Groningen, Oude Boteringestraat 52, Groningen, 9712 GL, The Netherlands; 3 Department of Health Care Policy, Harvard Medical School, 180 Longwood Avenue, Boston, Massachusetts, 02115, USA

**Keywords:** Major depressive disorder, subtypes, depressive symptoms, latent factor analyses, latent class analyses

## Abstract

**Background:**

According to current classification systems, patients with major depressive disorder (MDD) may have very different combinations of symptoms. This symptomatic diversity hinders the progress of research into the causal mechanisms and treatment allocation. Theoretically founded subtypes of depression such as atypical, psychotic, and melancholic depression have limited clinical applicability. Data-driven analyses of symptom dimensions or subtypes of depression are scarce. In this systematic review, we examine the evidence for the existence of data-driven symptomatic subtypes of depression.

**Methods:**

We undertook a systematic literature search of MEDLINE, PsycINFO and Embase in May 2012. We included studies analyzing the depression criteria of the *Diagnostic and Statistical Manual of Mental Disorders*, fourth edition (DSM-IV) of adults with MDD in latent variable analyses.

**Results:**

In total, 1176 articles were retrieved, of which 20 satisfied the inclusion criteria. These reports described a total of 34 latent variable analyses: 6 confirmatory factor analyses, 6 exploratory factor analyses, 12 principal component analyses, and 10 latent class analyses. The latent class techniques distinguished 2 to 5 classes, which mainly reflected subgroups with different overall severity: 62 of 71 significant differences on symptom level were congruent with a latent class solution reflecting severity. The latent class techniques did not consistently identify specific symptom clusters. Latent factor techniques mostly found a factor explaining the variance in the symptoms depressed mood and interest loss (11 of 13 analyses), often complemented by psychomotor retardation or fatigue (8 of 11 analyses). However, differences in found factors and classes were substantial.

**Conclusions:**

The studies performed to date do not provide conclusive evidence for the existence of depressive symptom dimensions or symptomatic subtypes. The wide diversity of identified factors and classes might result either from the absence of patterns to be found, or from the theoretical and modeling choices preceding analysis.

## Background

Major depressive disorder (MDD) is one of the most important challenges in global mental health [1,2]. In research, a continuing challenge is the diversity in the symptoms and pathophysiology of patients classified as having the disorder. MDD patients vary considerably in clinical presentation, course, treatment response, genetics and neurobiology [3-7]. One explanation for this diversity is that MDD has a polythetic definition; that is, a patient needs to satisfy some but not all symptoms. For the diagnosis at least five of nine symptoms including at least one of the two core symptoms must be present [8]. It follows that there are 227 possible combinations of symptoms leading to this diagnosis. This is such a wide array of possibilities that two patients classified as having MDD might have only a single symptom in common. This diversity raises the question whether it makes sense for the purposes of comparisons in research to consider all the people who qualify for the diagnosis of MDD as having a single disorder.

To overcome the problem of symptom diversity, several attempts have been made to specify more homogenous subgroups within MDD. Subtypes have been proposed based on specific combinations of symptoms (for example, melancholic depression, psychotic depression), onset (seasonal affective disorder, postpartum, early versus late in life), course (single, recurrent, chronic), or severity [6]. Most subtyping schemes are based on pattern recognition and ordering using distinctions observed in clinical practice. For instance, 11 subtypes of MDD were proposed in the Research Diagnostic Criteria (RDC), the forerunner of the current *Diagnostic and Statistical Manual of Mental Disorders *(DSM), based on combinations of clinical characteristics, follow-up patterns, and findings from family studies [9-11]. However, the value of such distinctions has been called into question by the disappointing results of attempts to use these and subsequent subtyping distinctions in clinical practice [6,12,13].

A different approach to discern useful subtypes with similar symptom profiles would be one that is data-driven; that is, which uses any of several statistical techniques to recognize patterns in reported symptoms of a heterogeneous group of subjects. These kinds of models have in common the fact that they reduce a large number of data from individuals to smaller numbers of latent variables based on similarity. Two dominant types of latent variable models are latent factor models and latent class models [14]. Latent factor models, such as exploratory factor analysis (EFA), reduce originally correlated variables to fewer latent factors (which might be specified as either correlated or uncorrelated) based on the correlations between the original variables [15,16]. By contrast, latent class models, such as cluster analysis (CA) and latent class analysis (LCA) cluster individuals rather than variables into relatively homogeneous subgroups. These subgroups are based on measures of similarity between each pair of individuals summed across all the variables considered in the analysis [17]. As both types of models are designed to discover structure in the absence of pre-existing hypotheses about subtypes, they provide useful approaches for examining heterogeneity based on distinctions that are not known beforehand [14].

Both latent factor and latent class models have been used to study the possible existence of useful MDD subtypes. However, findings in patients with MDD have not been systematically reviewed and thus the overall outcome is currently unclear. One of the questions, for instance, is whether a two-factor model applies to patients with MDD, as has been repeatedly found in patients with somatic illnesses. In somatically ill patients, latent factor analyses identified two main dimensions of depressive symptoms: one factor consisting of depressed mood, loss of interest, worthlessness, concentration problems and suicidality, and a second factor consisting of fatigue, appetite, sleep, and psychomotor disturbances [18,19]. The first set of symptoms is typically referred to as 'cognitive' or 'cognitive/affective', whereas the second set is typically referred to as 'somatic' or 'somatic/affective'; however, whether a cognitive and somatic symptom dimension is generalizable to patients with MDD or is limited to patients with somatic illnesses and comorbid depressive complaints is unknown. Systematic reviews or meta-analyses of studies empirically investigating the depressive symptom profiles of patients with MDD have not yet been performed.

In search of data-driven subtypes of MDD, we performed a systematic review of published studies that used these latent variable models to distinguish symptomatic subgroups or symptom dimensions for patients with MDD. Our main question was whether those studies identify consistent subtypes or symptom dimensions. Second, we evaluated whether the characteristic symptoms of empirically derived subtypes resemble the descriptions of current specifiers as melancholic and atypical [8]. Third, we studied whether latent factor analyses in patients with depression reveals a cognitive and somatic symptom dimension, as has been found in patients with somatic illnesses.

## Methods

### Search strategy

Studies were eligible for inclusion if they examined the existence of MDD subtypes by means of a latent variable analysis of depressive symptoms in patients with MDD. We searched three electronic databases MEDLINE (PubMed), Embase and PsycINFO for studies up to May 2012. We used the keywords 'major depressive disorder' and several synonyms for depressive subtype, symptom profile or symptom cluster (for full search strings, see Additional file 1). Many terms were used in the search, and were tested repeatedly for their success in finding relevant papers, based on our discovery of considerable variation in terminology across relevant articles. We used the database filters to exclude animal studies and studies with children. We did not use language restrictions. Retrieved articles were supplemented by studies cited in the reference lists of included studies plus a limited number of articles we found by hand searching.

### Inclusion criteria

An article was eligible if it presented original data. As our primary interest was adult patients with MDD, we required that at least 75% of the studied subjects had to satisfy the criteria for MDD. We did not use a stricter criterion of 100% patients with MDD, because for reasons of completeness, we did not want to exclude studies with a minor percentage of patients with minor depression, adjustment disorder, or dysthymia. We included studies classifying patients using the criteria of the RDC, DSM (III or later versions), International Classification of Diseases (ICD; 9 or 10), and Geriatric Mental State-Automated Geriatric Examination for Computer Assisted Taxonomy (GMS-AGECAT) systems, based on the fact that the core symptoms of MDD in these different systems have a great deal of overlap [9,20-25]. To limit the diversity in the patient group, we excluded studies focusing on somatically ill patients with comorbid MDD. We selected all studies analyzing the existence of symptomatic subtypes by means of a latent variable statistical method. We were interested in all statistical methods capable of finding symptom dimensions or latent classes in the depressive symptoms. With regard to those symptoms, we organized results by the nine Criterion A symptoms of depression in the DSM-IV text revision, but with dichotomous distinctions made for the three compound psychophysiological symptoms in that diagnostic system (s3 (appetite/weight disturbance), s4 (sleep disturbance) and s5 (psychomotor disturbance)). These dichotomous distinctions (for example, between insomnia and hypersomnia) might have value in distinguishing subtypes [4] such as the previously suggested subtypes of 'typical' and 'atypical' depression [8]. Studies were included in our review if they performed a latent variable analysis on at least 6 of the 12 resulting disaggregated symptomatic criteria for MDD.

### Data extraction

**Figure 1:**
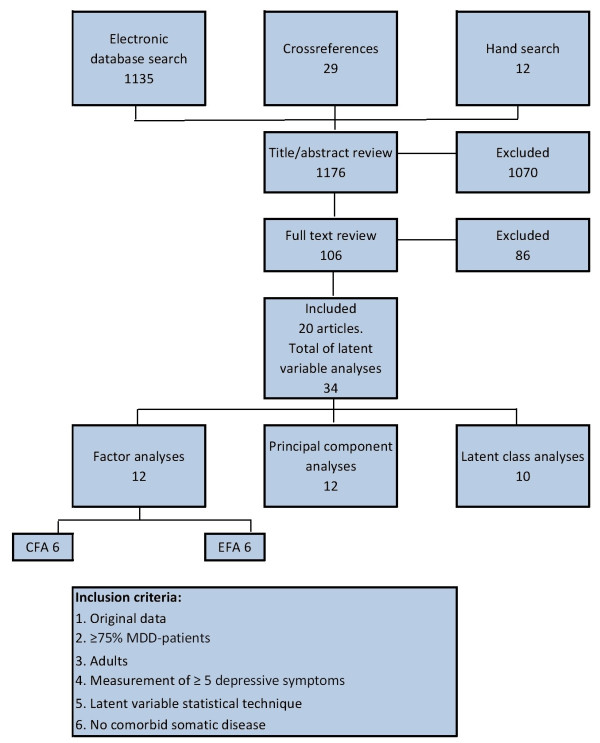
**Flow chart of the review**.

The database search described above resulted in 1,135 articles. Analysis of the reference lists of included articles provided another 29 relevant titles. Hand searching identified an additional 12 articles, giving a total of 1,176 unique titles. Subsequently, two independent raters (HMvL and PdJ reviewed the first half of articles, κ = 0.70; HMvL and RAS reviewed the second half of articles, κ = 0.80) reviewed the titles and abstracts of the identified studies to exclude any studies clearly not satisfying the inclusion criteria. If one (or both) of the reviewers assessed the title and abstract as possibly relevant, this article continued to be included in the review process. After this procedure, 93 studies remained included. We assessed the full text of all these 93 articles, and excluded 73 of them based on this review. In the end, 20 articles satisfied the inclusion criteria (see Additional file 2), containing 34 latent variable analyses in total (Figure 1).

### Data analysis

The 34 analyses were examined as follows. First, we extracted the study characteristics from the papers, and in some cases obtained supplementary information from the authors. Second, we examined all the different questionnaires used in the measurement of depressive symptoms (which included the Hamilton Rating Scale for Depression (HRSD), the Beck Depression Inventory (BDI), the Montgomery and Åsberg Depression Rating Scale (MADRS), the Inventory for Depressive Symptomatology (IDS), and the Brief Psychiatric Rating Scale (BPRS)). Using the original questionnaires, we determined which items of the different questionnaires corresponded to the 12 disaggregated depressive symptoms [26-34]. We then compared the results of the individual latent variable techniques. In the case of the latent factor techniques, we extracted the factors and the loadings of the 12 disaggregated symptoms on these factors. In the case of the latent class techniques, we extracted the reported latent classes and the differences in scores of the 12 symptoms in each class. The diversity in the questionnaires used precluded the performance of a mega-analysis or meta-analysis.

## Results

### Literature search

The 34 analyses in the 20 articles satisfying the inclusion criteria included 24 analyses that concerned the investigation of symptom dimensions by means of factor analyses (n = 12) [35-39] or principal component analyses (n = 12) [40-45], and 10 that concerned latent class analyses grouping a large number of individuals with depression into a smaller number of patient subgroups [42,45-53]. No analyses combined a latent factor approach with a LCA even though statistical techniques that allow this to be carried out do exist [14]. The total number of patients with depression was 7684. Samples included both men (38%) and women (62%), and both inpatients and outpatients. Overall, 96.6% of the study subjects satisfied the criteria for MDD. A small minority (3.4%) of patients has been given other diagnoses such as minor depressive disorder or adjustment disorder, which were found in 7 of 20 articles (see Additional file 3, Table S1 and Table S2).

### Measurement of depressive symptoms

In total, 11 different questionnaires (15 if we take into account the different versions of the HRSD, SCL, and IDS) were used to measure depressive symptoms in the 20 studies, whereas questionnaires measuring all 12 disaggregated symptoms were rare (3 of 11, which were the IDS; the SADS, Schedule for Affective Disorders and Schizophrenia; and the Structured Clinical Interview for DSM (SCID)). In particular, few questionnaires measured both directions of the so-called somatic symptoms (s3 to s6). For instance, data on appetite/weight gain (s3b) and hypersomnia (s4b) were lacking in the majority of analyses. Overall, of the 34 latent variable analyses, only 6 analyses included all 12 disaggregated depressive symptoms. In general, substantially different questions were used to measure the same symptoms. These differences were especially marked for symptoms of feeling worthless or guilty (s7) and having diminished ability to think or concentrate, or increased indecisiveness (s8). Answer categories were ordinal in 15 studies, binary in 3 studies (yes/no dichotomized), and mixed in 2 studies.

### Latent class analyses

**Figure 2:**
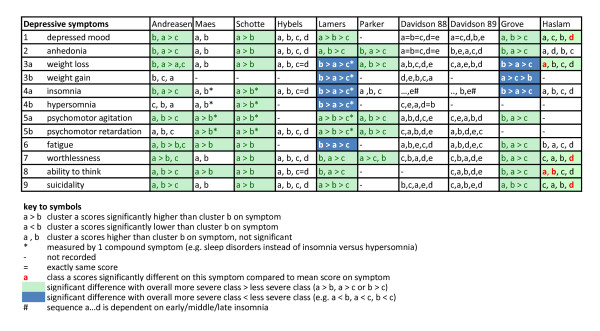
**Latent class analyses**.

**Figure 3:**
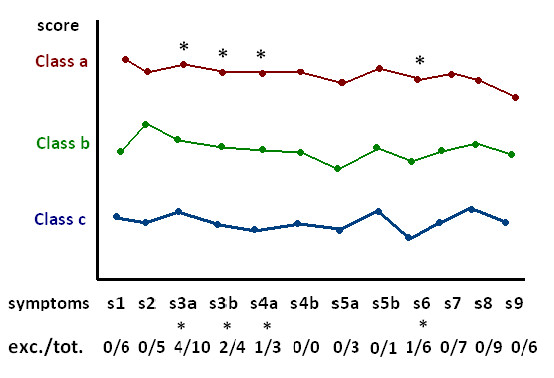
**General results from latent class analyses**. Abbreviations: tot., total significant differences in scores on disaggregated symptoms; exc., exceptions on ordering based on severity.

The 10 studies aimed at clustering patients based on symptom similarity comprised a total of 3,270 patients, with a mean of 327 patients analyzed per study (range 80 to 818). To visualize the effect of severity on class assignment, we sorted the original identified classes per study on overall severity. Therefore, we used the overall score of the class on each complete questionnaire, with class (a) being the most severe class of the study, class (b) being less severe than (a) and so on (see Additional file 3, Table S3). In total, there were 71 significant differences in symptom scores between classes. Notably, 62 of the 71 significant differences were related to class severity; that is, the overall most severe class scored higher on the symptom than the overall less severe class (Figure 2). This is in line with the idea that these statistical techniques are specifically able to separate more severe classes from less severe classes [14]. This pattern was especially clear in four of these studies [45-48]. Only nine significant differences deviated from this result [49,53] (Figure 3); all nine of these exceptions concerned so-called somatic depressive symptoms, and were present in latent class analyses of the two largest samples of patients (n = 569 and n = 818), in which patients in a less severe class scored higher than patients in a more severe class for fatigue (s6), weight (s3), and sleep disturbances (s4).

### Latent factor analyses

Our search yielded 24 analyses aimed at discovering dimensions of depressive symptoms. In total, there were 12 factor analyses and 12 principal component analyses (PCA) from 12 studies (some studies performed different analyses on the same patient sample). The factor analysis studies concerned six exploratory factor analyses (EFA) and six confirmatory factor analyses (CFA). The 12 factor analyses were performed on larger studies, with a mean of 562 patients per study (range 96 to 1049, total n = 3369). The 12 PCA concerned on average 174 patients (range 60 to 400, total n = 1045).

#### Confirmatory factor analyses

In two studies, CFA was used to test how well a single factor explained the variance in depressive symptoms [35,36] (Table 1). The measures for model fit (Confirmatory Fit Index, Tucker-Lewis Index, and Root Mean Square Error of Approximation) showed that a single factor explained about 50% of variance in depressive symptoms.

**Table 1:** Confirmatory factor analyses.

Author	Number of factors	Items in analysis	Explained variance	TLI	CFI	RMSEA	Factor correlation
Uher [12]	1	MADRS	0.57	0.99	0.97	0.10	NA
	1	HRSD-17	0.36	0.93	0.87	0.09	NA
	1	HRSD-6*	0.48	0.99	0.98	0.07	NA
	1	BDI	0.48	0.97	0.88	0.10	NA
	1	All 48 items	0.45	0.90	0.63	0.17	NA
Lux [35]	1	9 MDD symptoms	-	0.97	0.96	0.07	NA
	2	9 MDD symptoms	-	0.97	0.97	0.06	0.83

Abbreviations: BDI, Beck Depression Inventory; CFI, Confirmatory Fit Index; HRSD, Hamilton Rating Scale for Depression; MADRS, Montgomery and Åsberg Depression Rating Scale; MDD, major depressive disorder; NA, not applicable; * HRSD-6 contains 6 items out of the HRSD-17 (items 1,2,7,8,10,13) corresponding to depressive symptoms 1,2,5b,6,7; - not recorded.

#### Exploratory factor and principal component analyses

**Figure 4:**
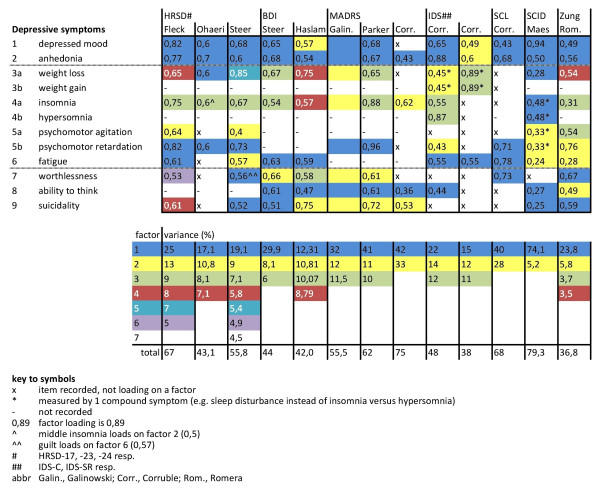
**Principal component and exploratory factor analyses**.

Nine studies exploratorively analyzed the dimensions underlying items of single questionnaires by means of PCA and EFA. The resulting factors of the 10 PCA and 3 EFA differed considerably (Figure 4). First, the number of derived factors varied from 2 to 7 (mean 3.5) explaining between 36.8% and 79.3% of total variance (mean 55%). Second, the item content of the identified factors varied substantially. Concerning the cognitive symptoms (s1, s2, s7, s8, s9) of patients with depression, 10 of 13 analyses included all 5 cognitive symptoms, but of those 10, there was not a single study in which all 5 cognitive symptoms loaded on the same factor. In 3 of the 10 studies, 4 of 5 cognitive symptoms loaded on the same factor [39,41,45], and in another 4 studies, 3 of the 5 cognitive symptoms loaded on the same factor [38,43,44]. Only one study had a factor on which four cognitive symptoms and no somatic symptoms loaded [39]; at least one somatic symptom loaded on the remaining six factors with three or more cognitive symptoms.

With regard to the somatic symptoms (s3 to s6), 8 of 13 studies measured the 4 somatic symptoms of depression, with at least one of the disaggregated symptoms of appetite/weight, sleep, and psychomotor disturbance included, and none of these 8 studies had a common factor on which all 4, or 3 of the 4, somatic symptoms loaded. In 7 out of 8 analyses, at least one factor was identified that explained the variance of 2 of the 4 somatic symptoms [39-41,44,45], and the total number of factors with 2 somatic symptoms was 10, describing the variance of 6 different pairs of somatic symptoms. Of those 10 factors with 2 somatic symptoms, 5 also incorporated one or more cognitive symptoms [37,39,40,44,45].

The most consistent finding was that the 2 core symptoms of depression (sadness and loss of interest; s1 and s2), loaded on one single factor in 11 out of 13 analyses [37-41,43-45]. In 9 of these 11 analyses, s1 and s2 loaded on factor 1, which was the factor explaining most of the variance. Psychomotor retardation and/or fatigue (s5b and s6) also loaded on the same factor in 8 of 11 analyses [38,40-44]. Thus, the most prevalent finding was one single factor explaining the variance of a mixture of cognitive and somatic symptoms (s1, s2, s5b, and s6).

**Figure 5:**
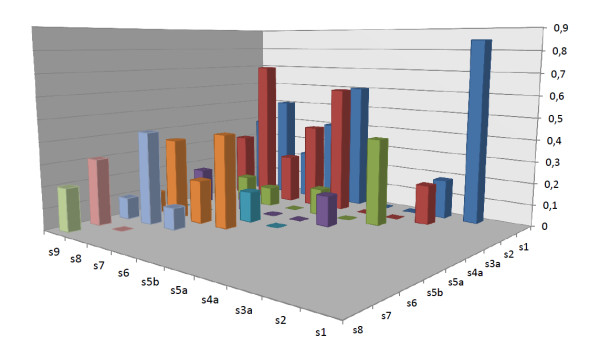
**Pairwise common factor loadings**. The x- and z-axes show disaggregated depressive symptoms, while the y-axis shows the proportion of pairwise common factor loadings.

Figure 4 shows the diversity in the retrieved factors, such as the number, the item content, and loadings. Figure 5 was derived from Figure 4, and shows the proportion of pairwise common factor loadings. In total, the studies described 461 measurements of pairs of symptoms (for example, the combination of s1 and s2 was measured in all 13 studies, while the combination of s1 and s5a was measured in 7 studies). Of these 461 measurements of symptom pairs, 97 pairs loaded on a common factor, resulting in an average proportion of pairwise common factor loadings of 0.21 (97/461). In Figure 5, the heights of the 45 vertical bars depend on the proportion of each pair of symptoms that loaded on a common factor; for example, pair s1 and s2 loaded 11 out of 13 times on a common factor, resulting in a proportion of 0.85 pairwise common factor loadings. In case of a clear two-factor solution, one would expect to find a landscape of two areas with high bars, and one area with low bars. Obviously this is not the case, and neither can those areas be found by rearranging the symptom orders on the x- or z-axis. Furthermore, for relatively many symptom pairs the proportion of common factor loadings approached the average of 0.21, which is not consistent with a clear two-factor solution. Of note, weight gain (s3b) and hypersomnia (s4b) were measured too infrequently to include in this figure.

**Figure 6:**
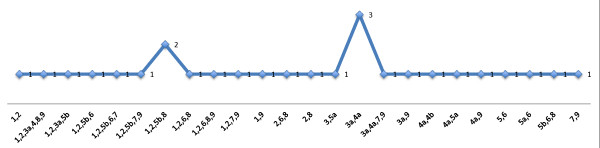
**Frequencies of symptom combinations loading on a common factor**. The x-axis shows all symptom combinations loading on a common factor, while the y-axis shows the number of times this specific common factor has been identified.

Figure 6 was also derived from Figure 4, and shows all depressive symptom combinations that were found to load on a common factor. In total, 27 factors had an item content of 2 or more depressive symptoms. The item content of those 27 factors was unique in 22 factors; only 5 factors had a non-unique item content, with two symptom combinations occurring more than once (Figure 6).

#### Influence of different patient samples (same variables)

Several studies analyzed the same questionnaires with different patient samples: three studies used the HRSD [37,40,41], two studies the BDI [41,42] and three studies the MADRS [38,43,44]. The analyses of the same questionnaires resulted in substantial different factors, both qualitatively (item content) and quantitatively (number of factors). Varying influences of the patient sample on the resulting dimensional structure can be seen from the collected analyses (Figure 4). First, differences in severity affected the factorial structure. Galinowski and Lehert performed two PCA of the MADRS from the same group of patients at a different timepoint [43]. On day 0, before antidepressant treatment, three components explained 55.5% of the variance of the MADRS items, whereas after 28 days of antidepressant treatment, one component explained 66% of the variance. A likely explanation is the diminished overall severity score on the MADRS (from 35 to 17 points), with a decrease in correlations between items. However, such severity differences do not explain all variety in factor structure. This is illustrated by the studies of Corruble *et al*. and of Galinowski and Lehert [43,44], which both had a patient sample with a roughly similar mean MADRS score (34 vs. 35 points), yet, the principal components derived from the samples differ considerably (two versus three PCs, explaining 75% vs. 55.5% of the variance with a different item content). Similar diverging factor patterns resulted from the studies analyzing the BDI or HRSD.

#### Influence of different questionnaires (same patient sample)

Two studies investigated the factor structure of different questionnaires from the same patient sample in separate analyses. First, Steer *et al*. performed two PCA on the BDI and the HRSD, which resulted in three and seven qualitatively different components, respectively [41](Figure 4). Second, Corruble *et al*. performed four PCA on the IDS-C, IDS-SR, MADRS and the SCL-90R [44], and the variety in the resulting factor structure was again substantial. Even the two PCA on the IDS Clinician (IDS-C) and IDS Self-Report (IDS-SR) ratings produced components with a different item content, though the questionnaires are largely similar.

#### Combining items of multiple questionnaires in one analysis

Four studies performed latent factor analyses on a combination of items from different questionnaires [36,37,41,54]. Although response categories vary between different questionnaires, no study used a smoothing method to adjust for these variances, which could explain part of the following results. Steer *et al*. performed PCA on a combination of all HRSD and BDI items. All BDI items loaded on principal component 1 (PC1), whereas the HRSD items loaded on PC2 and PC3 [41]. A largely similar result was found by Uher *et al*., using EFA on 47 items from the MADRS, the HRSD, and the BDI. The depressive symptom items of the MADRS and the HRSD predominantly loaded on factor 1, whereas BDI items mainly loaded on factor 3 [36]. Thus, those studies show a high correlation of items in one questionnaire. The opposite result was found by Gullion and Rush [54], who performed EFA on 88 items of the IDS-C, IDS-SR, HRSD, and BDI. They identified 10 factors mainly explaining the variance of items measuring the same symptom in the different questionnaires; for example, factor 2 comprised several items measuring guilt/worthlessness (s7), for instance 'guilt feelings' (BDI), 'guilt feelings' (HRSD), 'self-criticism and blame' (IDS-SR, IDS-C).

Ohaeri and Otote performed the fourth analysis in which items of different questionnaires are included, that is, items of the HRSD and the BPRS [37]. Of note, these authors also performed EFA on the HRSD items alone (Figure 4), therefore, the effect of the extra BPRS items on the factorial structure is directly observable in this study. First, EFA of the HRSD items exclusively resulted in four factors, explaining 43.1% of the variance. The first factor comprised six items: depressed mood, anhedonia, weight and appetite loss, psychomotor retardation, and paranoid symptoms, and this factor is described as 'core depressive'. Second, an EFA on the HRSD plus BPRS items also resulted in four factors, explaining 43.7% of the variance. The first factor is again described as 'core depressive'; however, this factor now contains different items. Of the previous six HRSD items only two remained: depressed mood and psychomotor retardation. By contrast, the item with the highest factor loading was uncooperativeness (0.8). In addition, the factor contained hypochondriasis, insight loss, and emotional withdrawal. A comparable shift in item content was found for the other factors.

## Discussion

Our results show that studies performed to date provide insufficient evidence to confirm the stable existence of clear data-driven symptomatic subtypes of depression. First, relatively few studies have been dedicated to the detection of data-driven MDD subtypes (20 of 1176 articles). Second, the outcomes of these few studies are conflicting. Latent class analyses mainly grouped patients on overall severity, but not in classes with qualitatively different symptom profiles, whereas latent factor analyses, most consistently identified a factor explaining the variance of a mixture of cognitive and somatic symptoms (s1, s2, s5b, s6), which seems in contradiction with a purely cognitive or somatic symptom dimension. However, the 13 identified factors differed to such an extent that generalizable conclusions are questionable. In short, the collected studies fail to give adequate evidence for the existence of qualitatively different subtypes or symptom dimensions of MDD. Thus, this lack of empirical support also holds for theoretical motivated subtypes such as as melancholic or atypical, or cognitive and somatic symptom dimensions in MDD.

Particularly notable is the great deal of diversity in the results. How can we explain this large diversity? From the collected results, it can be seen that all sorts of factors influence the outcomes of latent variable analyses; for instance, the included number of patients, and the severity and quantity of their symptoms obviously affect the resulting latent classes and factors. Presumably, there is considerable difference in symptom endorsement rates between the studies, as was reported from a recent mega-analysis of genome-wide association studies of patients with MDD [55]. Furthermore, the extensive number and differences of the questionnaires used, including all not recorded symptoms (weight gain, hypersomnia), is a likely contributor to the diverging results. On top of that, previous studies have found that in case of latent factor analyses, several items, including model choice (for example, component or common factor model), sample size, number and communalities of the variables, selection of the number of factors and model fit criteria, rotation method, and the degree of overdetermination, affect the stability and correspondence of the resulting factors [56-58]. An extra influence to take into account is the manifestly high correlation of symptoms within one questionnaire, as shown by some (not all) latent factor analyses on different questionnaires simultaneously. Thus, many theoretical choices preceding analysis are important determinants of the retrieved classes or dimensions.

The major insight from this review is that we should improve our research techniques considerably to find data-driven subtypes of depression. Of course, it is an open-ended question as to whether such a pattern exists. It is possible that there simply are no symptomatic subtypes or dimensions, and that latent variable techniques have failed to show consistent subtypes because of this fact. One could argue that if there were clear symptomatic subtypes or dimensions, a more consistent pattern would have emerged out of the data, regardless of the theoretical and measurement choices involved. The other possibility is that symptomatic subtypes and dimensions do exist, but that the techniques used to date did not succeed in identifying them. If the second possibility is true, a crucial question is how study methods could be improved to detect patterns in the data that have not yet been detected. Careful consideration of all theoretical and modeling choices that influence study outcomes will be required to answer that question.

One possible strategy to improve study methods is data enrichment, because it is clear that the quality of data crucially determines the quality of study outcomes. First, some of the studies reviewed above indicate the possible benefit of dynamic measurements, as they showed that differentiated symptom profiles might be clearer with more rather than less severe cases or for patients earlier rather than later in the treatment process. Therefore it would seem prudent to study changes in symptom structure and severity over the course of treatment, preferably also distinguishing the influence of medication. A second set of choices would involve the scales used to assess these symptoms. Some diagnostic instruments use dichotomous (yes/no) measures for each symptom, whereas others have gradual assessments. Gradual assessments, would of course be expected to provide textured differentiation, and in this way possibly lead to greater precision in detecting meaningful subtypes. Another concern is that the frequently used rating scales are primarily designed to be sensitive to change as opposed to capturing the detailed phenomenological picture of MDD. To address the potential heterogeneity in patients with depression, at least all DSM criteria, including all disaggregated symptoms (s3-s5), should be measured in a standardized fashion. A third possibility to enrich data would be the inclusion of other variables in addition to depressive symptoms in analyses. Concerning the symptoms to include in the evaluation, it seems clear that this set of symptoms should be a broad one, so as to allow for the possibility of detecting subtypes associated with symptoms beyond those in the current DSM and ICD systems. For example, there is some suggestion in the literature that there might be value in differentiating irritable from non-irritable MDD [59] and in assessing the influence of anxiety [60,61]. Moreover, apart from symptoms, other indicators, such as hormone status, genetic profile, sex, and age, could also be included as variables in future analyses. The inclusion of these non-symptomatic variables possibly contributes to the unraveling of causal mechanisms of depressive subtypes, although we firmly believe that symptoms are the basis on which to start. Symptoms are non-invasive measures of disease; clinicians are trained to recognize and classify patients based on symptoms; and data on symptoms have been collected world-wide.

A second strategy to improve study methods involves the statistical approaches used to uncover depressive dimensions and subtypes. In addition to the latent factor and latent class approaches, we should consider complex CA models, especially those that use a canonical formulation to predict diverse outcomes [62,63], and mixture models that combine features of latent class and item response theory models [64] or latent class and latent factor models [14]. Some recent studies have used factor mixture analyses to identify subtypes in other psychiatric disorders, such as attention-deficit/hyperactivity disorder [65], post-traumatic stress disorder [13], and schizophrenia [66]. To date, these approaches have not yet been used to search for subtypes of MDD, but are attractive alternatives in light of their success in detecting useful subtypes of other disorders. Obviously, in accordance with the described latent variable models, many theoretical factors should be considered before applying those new techniques [67]. However, if subtypes of MDD do exist, using different statistical methods to reveal their structure could be worthwhile.

Thus, future analyses should ideally explore several advanced statistical techniques on enriched datasets. An investigation of the possibilities and limitations of different modeling techniques seems more reasonable than adhering exclusively to the latent factor and latent class models used up to now. Mega-analyses of the MDD symptoms of different samples could be worthwhile, as combined data may have a positive effect on robustness and generalizability of the results [55,68]. However, when performing mega-analyses, it is even more important to have rich datasets and to apply sophisticated modeling techniques to the data to accommodate inter-study heterogeneity [55]. Experiences with those new symptom-based classification attempts might inform other data-driven classification attempts that go beyond the DSM, such as the Research Domain Criteria [69,70].

Finally, what is the value of searching for data-driven subtypes of MDD? We started our review with the observation that patients with MDD differ considerably in their symptomatic presentation, with over 200 possible symptom combinations. To date, theoretical motivated subtypes have not resolved the substantial population heterogeneity of MDD. Empirical discernment of subtypes with similar symptoms could give an impetus to research on etiology, course, and treatment. Improved statistical tools are available to discover patterns in rich datasets, therefore, data-driven subtyping of depression is a valuable approach to be explored.

## Conclusions

There is no conclusive evidence for the existence of depressive symptom dimensions or symptomatic subtypes in adults with MDD. Many theoretical and modeling choices affect the results of latent variable analyses. If there is any structure to be discovered in the current heterogeneity, consideration of those choices would provide an useful starting point for future studies.

## Abbreviations

BDI: Beck Depression Inventory; BPRS: Brief Psychiatric Rating Scale; CA: cluster analysis; CFA: confirmatory factor analysis; DSM: Diagnostic and Statistical Manual of Mental Disorders; EFA: exploratory factor analysis; GMS-AGECAT: Geriatric Mental State - Automated Geriatric Examination for Computer Assisted Taxonomy; HRSD: Hamilton Rating Scale for Depression; ICD: International Classification of Diseases; IDS (-C/-SR): Inventory for Depressive Symptomatology (Clinician/Self-Report); LCA: latent class analysis; MADRS: Montgomery and Åsberg Depression Rating Scale; MDD: major depressive disorder; PC: principal component; PCA: principal component analysis; RDC: Research Diagnostic Criteria; RMSEA: Root Mean Square Error of Approximation; SCL: Symptom Checklist; TLI: Tucker-Lewis Index; ZSDS: Zung Self-rating Depression Scale.

## Competing interests

The authors declare no competing interests.

## Authors' contributions

The study was designed by HMvL, PdJ and RAS. HMvL conducted the literature search. HMvL, PdJ and RAS assessed the eligibility of the studies for inclusion. HMvL extracted data from the included papers, and drafted the manuscript. JWR contributed to the interpretation of the results. PdJ, JWR, RCK, and RAS critically assessed drafts of the manuscript. All authors read and approved the final manuscript.

## Authors' information

HMvL is a medical doctor and doctoral scholar at the University Center for Psychiatry (UCP), University Medical Center Groningen (UMCG), the Netherlands. PdJ is a professor at the UCP, UMCG and Center of Research on Psychology in Somatic Diseases at Tilburg University. JWR is professor at the Faculty of Philosophy of the University of Groningen. RCK is McNeil Family Professor of Health Care Policy at the Department of Health Care Policy at Harvard Medical School. RAS is professor and head of the UCP, UMCG.

## Pre-publication history

The pre-publication history for this paper can be accessed here:

http://www.biomedcentral.com/1741-7015/10/156/prepub

## Supplementary Material

Additional file 1 **Search strings**. The search strings and results of the electronic database search. Click here for file

Additional file 2 **References of included studies**. The references of the 20 studies included in this systematic review. Click here for file

Additional file 3 **Study characteristics**. Study characteristics of the included latent class analyses (Table S1), and latent factor analyses (Table S2), including the translation of the original clusters identified by latent class analyses to a class ordering based on severity (Table S3). Click here for file
