# Supplementary material for: Data-driven subtypes of major depressive disorder: a systematic review
Source: BMC Med. 2012 Dec 4;10:156. doi: 10.1186/1741-7015-10-156 (PMC3566979; doi:10.1186/1741-7015-10-156)
Supplement: Additional file 1 — Search strings. The search strings and results of the electronic database search. [file 1741-7015-10-156-S1.PDF]

**SEARCH STRINGS**

| Database | MDD | Outcome | Limits | Total |
|---|---|---|---|---|
| **Pubmed** | "Depressive Disorder, Major"[Majr] OR (major depressive disorder*[tiab] OR major depression*[tiab] | depressive symptom cluster* [tiab] OR depressive subtype*[tiab] OR depressed patient type*[tiab] OR subtypes of unipolar depression*[tiab] OR subtypes of depression*[tiab] OR types of depression*[tiab] OR types of depressive disorder*[tiab] OR depressive typ*[tiab] OR subtyping depression*[tiab] OR subtype of major depressi*[tiab] OR subtypes of major depressi*[tiab] OR subtyping depressive disorder*[tiab] OR subtyping major depressi*[tiab] OR profiles of depressive symptom*[tiab] OR symptomatic criteria[tiab] | Human, Adult | 833 |
| **Embase** | 'major depression'/exp/mj OR 'major depression':ab,ti OR 'major depressive disorder':ab,ti AND [embase]/lim | 'depressive symptom cluster':ab,ti OR 'depressive symptom clusters':ab,ti OR 'depressive subtype':ab,ti OR 'depressive subtypes':ab,ti OR 'depressed patient type':ab,ti OR 'depressed patient types':ab,ti OR 'subtypes of unipolar depression':ab,ti OR 'subtypes of depression':ab,ti OR 'types of depression':ab,ti OR 'types of depressive disorder':ab,ti OR 'depressive type':ab,ti OR 'subtyping depression':ab,ti OR 'subtypes of major depression':ab,ti OR 'subtyping depressive disorder':ab,ti OR 'subtyping major depressive disorder':ab,ti OR 'profiles of depressive symptoms':ab,ti OR 'symptomatic criteria':ab,ti AND [embase]/lim | Embase, Human, Adult | 149 |
| **PsycINFO** | MM "Major Depression" or TI "major depressive disorder*" or AB "major depressive disorder*" or TI "major depression*" or AB "major depression*" | TI "depressive subtyp*" or AB "depressive subtyp*" or TI "depressive symptom cluster*" or AB "depressive symptom cluster*" or TI "depressed patient typ*" or AB "depressed patient typ*" OR TI "subtypes of depressi*" OR AB "subtypes of depressi*" OR TI "types of depressi*" OR AB "types of depressi*" OR TI "depressive typ*" OR AB "depressive typ*" OR TI "subtyping depressi*" OR AB "subtyping depressi*" OR TI "profiles of depressive symptom*" OR AB "profiles of depressive symptom*" OR TI "subtypes of unipolar depressi*" OR AB "subtypes of unipolar depressi*" OR TI "subtypes of major depressi*" OR AB "subtypes of major depressi*" OR TI "subtyping major depressi*" OR AB "subtyping major depressi*" OR TI "symptomatic criteria" OR AB "symptomatic criteria” | Human, Adult | 400 |

Total identified references by electronic database search: 1382. After removal of duplicates: 1135 unique titles to be included in the review process.
