# Supplementary material for: Data-driven subtypes of major depressive disorder: a systematic review
Source: BMC Med. 2012 Dec 4;10:156. doi: 10.1186/1741-7015-10-156 (PMC3566979; doi:10.1186/1741-7015-10-156)
Supplement: Additional file 2 — References of included studies. The references of the 20 studies included in this systematic review. [file 1741-7015-10-156-S2.PDF]

REFERENCES OF INCLUDED STUDIES

Andreasen NC, Grove WM: **The classification of depression: Traditional views versus mathematical approaches.** Am J Psychiatry 1982, **139**(1):45-52.

Corruble E, Legrand JM, Duret C, Charles G, Guelfi JD: **IDS-C and IDS-SR: Psychometric properties in depressed in-patients.** J Affect Disord 1999, **56**(2-3):95-101.

Davidson J, Woodbury MA, Pelton S, Krishnan KR: **A study of depressive typologies using grade of membership analysis.** Psychol Med 1988, **18**(1):179-189.

Davidson JR, Woodbury MA, Zisook S, Giller EL: **Classification of depression by grade of membership: A confirmation study.** Psychol Med 1989, **19**(4):987-998.

Fleck MPdA, Poirier-Littre M, Guelfi J-, Bourdel MC: **Factorial structure of the 17-item Hamilton Depression Rating Scale.** Acta Psychiatr Scand 1995, **92**(3):168-172.

Galinowski A, Lehert P: **Structural validity of MADRS during antidepressant treatment.** Int Clin Psychopharmacol 1995, **10**(3):157-161.

Grove WM, Andreasen NC, Young M, Endicott J: **Isolation and characterization of a nuclear depressive syndrome.** Psychol Med 1987, **17**(2):471-484.

Gullion CM, Rush AJ: **Toward a generalizable model of symptoms in major depressive disorder.** Biol Psychiatry 1998, **44**(10):959-972.

Haslam N, Beck AT: **Categorization of major depression in an outpatient sample.** J Nerv Ment Dis 1993, **181**(12):725-731.

Hybels CF, Blazer DG, Pieper CF, Landerman LR, Steffens DC: **Profiles of depressive symptoms in older adults diagnosed with major depression: Latent cluster analysis.** Am J Geriatr Psychiatry 2009, **17**(5):387-396.

Lamers F, De Jonge P, Nolen WA, Smit JH, Zitman FG, Beekman ATF, Penninx BWJH: **Identifying depressive subtypes in a large cohort study: Results from the Netherlands Study of Depression and Anxiety (NESDA).** J Clin Psychiatry 2010, **71**(12):1582-1589.

Lux V, Aggen SH, Kendler KS: **The DSM-IV definition of severity of major depression: inter-relationship and validity.** Psychol Med 2010, **40**(10):1691-1701.

Maes M, Maes L, Schotte C, Cosyns P: **A clinical and biological validation of the DSM-III melancholia diagnosis in men: Results of pattern recognition methods.** J Psychiatr Res 1992, **26**(3):183-196.

Ohaeri JU, Otote DI: **Family history, life events and the factorial structure of depression in a Nigerian sample of inpatients.** Psychopathology 2002, **35**(4):210-219.

Parker G, Wilhelm K, Mitchell P, Roy K, Hadzi-Pavlovic D: **Subtyping depression: Testing algorithms and identification of a tiered model.** J Nerv Ment Dis 1999, **187**(10):610-617.

Parker RD, Flint EP, Bosworth HB, Pieper CF, Steffens DC: **A three-factor analytic model of the MADRS in geriatric depression.** Int J Geriatr Psychiatry 2003, **18**(1):73-77.

Romera I, Delgado-Cohen H, Perez T, Caballero L, Gilaberte I: **Factor analysis of the Zung self-rating depression scale in a large sample of patients with major depressive disorder in primary care.** BMC Psychiatry 2008, **8**:4.

Schotte CKW, Maes M, Cluydts R, Cosyns P: **Cluster analytic validation of the DSM melancholic depression. The threshold model: Integration of quantitative and qualitative distinctions between unipolar depressive subtypes.** Psychiatry Res 1997, **71**(3):181-195.

Steer RA, Beck AT, Riskind JH, Brown G: **Relationships between the Beck Depression Inventory and the Hamilton Psychiatric Rating Scale for Depression in depressed outpatients.** Journal of Psychopathology and Behavioral Assessment 1987, **9**(3):327-339.

Uher R, Farmer A, Maier W, Rietschel M, Hauser J, Marusic A, Mors O, Elkin A, Williamson RJ, Schmael C, Henigsberg N, Perez J, Mendlewicz J, Janzing JGE, Zobel A, Skibinska M, Kozel D, Stamp AS, Bajs M, Placentino A, Barreto M, McGuffin P, Aitchison KJ: **Measuring depression: Comparison and integration of three scales in the GENDEP study.** Psychol Med 2008, **38**(2):289-300.
