# Supplementary material for: Data-driven subtypes of major depressive disorder: a systematic review
Source: BMC Med. 2012 Dec 4;10:156. doi: 10.1186/1741-7015-10-156 (PMC3566979; doi:10.1186/1741-7015-10-156)
Supplement: Additional file 3 — Study characteristics. Study characteristics of the included latent class analyses (Table S1), and latent factor analyses (Table S2), including the translation of the original clusters identified by latent class analyses to a class ordering based on severity (Table S3). [file 1741-7015-10-156-S3.PDF]

STUDY CHARACTERISTICS

Table S1. Latent class analyses

	Andreasen	Maes	Schotte	Hybels	Lamers	Park. 99	Dav. 88	Dav. 89	Grove	Haslam
**N**	228	80	220	366	818	269	190	130	569	400
**MDD diagnosis**	RDC	DSM III R	DSM III	DSM IV	DSM IV	DSM III R	RDC	RDC	-	DSM III
**% no MDD**	23	24	25	0	9,2	0	6,3	12	0	0
**Male / female**	94 / 134	80 / 0	51 / 169	124 / 242	272 / 546	-	56 / 136	69 / 61	214 / 298	146 / 254
**Country**	USA	Belgium	Belgium	USA	NL	Australia	USA	USA	USA	USA
**Mean severity**	-	HDRS-21<br>22,3	HDRS-17<br>19,32	MADRS<br>26,82	-	-	HDRS-25<br>28,1	HDRS-25<br>34,9	-	BDI<br>27,3
**Method**	CA	CA	CA	LCCA	LCA	LCA	GOM	GOM	CA, LCA	COBWEB3
**Items in analysis**	106 SADS	14 SCID	14 SCID	10<br>MADRS	10 CIDI<br>6 IDS-SR	16 clinical<br>features	47 of HRSD<br>SCL-90<br>Newcastle	HRSD<br>SCL-58<br>CDC	36 SADS	21 BDI
**Structure of items**	Ordinal	Binary	Binary	Ordinal	Binary	Binary +<br>ordinal	Ordinal	Binary +<br>ordinal	Ordinal	Ordinal

Table S2. Latent factor analyses

	Fleck	Ohaeri	Steer	Haslam	Galin.	Park. 03	Corruble	Maes	Romera	Gullion	Lux	Uher
**N**	60	96	300	400	137	225	68	80	1049	324	1015	660
**MDD diagnosis**	DSM III R	ICD 10	DSM III	DSM III	DSM III	DSM IV	DSM III R	DSM III R	DSM IV	DSM III R	DSM IV	DSM IV
**% no MDD**	15	16	0	0	0	0	0	24	0	0	0	0
**Male / female**	14 / 46	31 / 65	109 / 191	146 / 254	64 / 36	-	23 / 45	80 / 0	282 / 856	105 / 217	518 / 497	236 / 424
**Country**	France	Nigeria	USA	-	-	USA	-	Belgium	Spain	USA	USA	Europe
**Mean severity**	HDRS-17<br>26,6	-	HDRS-24<br>21,24	BDI<br>27,3	MADRS<br>34,76	-	MADRS<br>33,9	HDRS-24<br>22,3	Zung<br>54,13	HRSD-17<br>18,8	-	HRSD-17<br>13,61
**Method**	PCA	EFA	PCA	PCA	PCA	EFA	PCA	PCA	EFA	EFA	CFA	CFA
**Items in analysis**	HDRS-17	HDRS-23<br>BPRS-18	HDRS-24<br>BDI	BDI	MADRS	MADRS	MADRS<br>IDS-C/-SR<br>SCL-90R	SCID	Zung	HRSD-17<br>IDS-C/-SR<br>BDI	9 DSM-<br>criteria<br>for MDD	MADRS<br>HRSD-17<br>BDI
**Structure of items**	Ordinal	Ordinal	Ordinal	Ordinal	Ordinal	Ordinal	Ordinal	Binary	Ordinal	Ordinal	Ordinal	Ordinal

Abbreviations
CA, cluster analysis; EFA, exploratory factor analysis; GOM, grade of membership analysis; NL, the Netherlands;
LCCA, latent class cluster analysis; LCA, latent class analysis; PCA, principal component analysis.

LATENT CLASS ANALYSES

Table S3. Translation of original clusters to severity based classes

Severity based	Andreasen	Maes	Schotte	Hybels	Lamers	Park. 99	Dav. 88	Dav. 89	Grove	Haslam
**Class a**	cluster 4<br>n=10<br>∑ 59,9	cluster 2<br>n=35<br>∑ 26,9	cluster 1<br>n=99<br>∑ 22,9	cluster 4<br>n=25<br>∑ 35,7	cluster 1<br>n=379<br>p* 0,8	cluster 1<br>n=30<br>∑ 18,6	cluster 5<br>n=24<br>∑ 38,4	cluster 5<br>n=23<br>∑ 41,5	cluster 3<br>n=57<br>∑ 77,0	cluster 2<br>n=161<br>∑ 28,6
**Class b**	cluster 1<br>n=99<br>∑ 56,5	cluster 1<br>n=45<br>∑ 18,7	cluster 2<br>n=121<br>∑16,4	cluster 2<br>n=99<br>∑ 24,6	cluster 2<br>n=201<br>p* 0,81	cluster 2<br>n=91<br>∑ 10,0	cluster 4<br>n=37<br>∑ 34,1	cluster 2<br>n=25<br>∑ 40,9	cluster 1<br>n=248<br>∑ 75,1	cluster 1<br>n=166<br>∑ 27,6
**Class c**	cluster 2<br>n=67<br>∑ 45,5			cluster 1<br>n=173<br>∑ 19,2	cluster 3<br>n=238<br>p* 0,64	cluster 3<br>n=148<br>∑ 1,7	cluster 2<br>n=51<br>∑ 28,3	cluster 4<br>n=27<br>∑ 39,4	cluster 2<br>n=248<br>∑ 60,6	cluster 3<br>n=41<br>∑ 26,3
**Class d**				cluster 4<br>n=69<br>∑ 15,8			cluster 1<br>n=31<br>∑ 23,0	cluster 3<br>n=29<br>∑ 30,4		cluster 4<br>n=32<br>∑ 20,4
**Class e**							cluster 3<br>n=47<br>∑ 21,2	cluster 1<br>n=26<br>∑ 23,8		
**∑ : sum score of**	SADS<br>16 items	HRSD-21	HRSD-17	MADRS	CIDI*	CORE	HRSD-25	HRSD-25	SADS<br>36 items	BDI

* Average probability of symptom endorsement on 10 CIDI depressive items.
